# ReGeNNe: genetic pathway-based deep neural network using canonical correlation regularizer for disease prediction

**DOI:** 10.1093/bioinformatics/btad679

**Published:** 2023-11-14

**Authors:** Divya Sharma, Wei Xu

**Affiliations:** Biostatistics Department, Princess Margaret Cancer Center, University Health Network, Toronto, ON M5G2C4, Canada; Division of Biostatistics, Dalla Lana School of Public Health, University of Toronto, Toronto, ON M5T 3M7, Canada; Biostatistics Department, Princess Margaret Cancer Center, University Health Network, Toronto, ON M5G2C4, Canada; Division of Biostatistics, Dalla Lana School of Public Health, University of Toronto, Toronto, ON M5T 3M7, Canada

## Abstract

**Motivation:**

Common human diseases result from the interplay of genes and their biologically associated pathways. Genetic pathway analyses provide more biological insight as compared to conventional gene-based analysis. In this article, we propose a framework combining genetic data into pathway structure and using an ensemble of convolutional neural networks (CNNs) along with a Canonical Correlation Regularizer layer for comprehensive prediction of disease risk. The novelty of our approach lies in our two-step framework: (i) utilizing the CNN’s effectiveness to extract the complex gene associations within individual genetic pathways and (ii) fusing features from ensemble of CNNs through Canonical Correlation Regularization layer to incorporate the interactions between pathways which share common genes. During prediction, we also address the important issues of interpretability of neural network models, and identifying the pathways and genes playing an important role in prediction.

**Results:**

Implementation of our methodology into three real cancer genetic datasets for different prediction tasks validates our model’s generalizability and robustness. Comparing with conventional models, our methodology provides consistently better performance with AUC improvement of 11% on predicting early/late-stage kidney cancer, 10% on predicting kidney versus liver cancer type and 7% on predicting survival status in ovarian cancer as compared to the next best conventional machine learning model. The robust performance of our deep learning algorithm indicates that disease prediction using neural networks in multiple functionally related genes across different pathways improves genetic data-based prediction and understanding molecular mechanisms of diseases.

**Availability and implementation:**

https://github.com/divya031090/ReGeNNe.

## 1 Introduction

Accurate diagnosis and prognosis of diseases requires the clinicians to analyze patient biopsies, imaging or blood samples for characteristic disease biomarkers including unregulated pathways or genes (Torkamani *et al.* 2008, Claussnitzer *et al.* 2020). Genetic discoveries have substantially improved our understanding of disease mechanisms and have made it feasible to develop methods to identify risk and therapeutic genes that could enable earlier diagnostic evaluation and better prevention and therapeutic strategies (Emilsson *et al.* 2008, Natarajan and Dhillon 2014).

Recent studies have explored diagnosis prediction through disease–gene and disease–pathway associations using machine learning methodologies (Yao *et al.* 2014, Kim *et al.* 2018, Zhang *et al.* 2018, Shi *et al.* 2021, Zeng *et al.* 2021). Dasgupta *et al.* (2011) propose a supervised machine learning disease prediction model to map the relationships between individual sample genotype data and the associated disease. Ata *et al.* (2021) review computational methods leveraging network/graph data for disease–gene prediction. Asif *et al.* (2018) use machine learning classifiers trained on gene functional similarities to identify genes involved in complex diseases. Elmarakeby *et al.* (2021) propose a deep learning model to classify patients with prostate cancer by treatment-resistance state and evaluate molecular drivers of treatment resistance. Abeel *et al.* (2010) propose support vector machines and combined feature selection methods for selection of gene biomarkers.

The rationale behind using gene pathways to understand disease mechanisms is firstly, genes/proteins do not work alone, but in an intricate network of interactions and pathways. In addition, complex diseases are more likely caused by the dysregulation of multiple targets in connected pathways or different genes in the same pathways in patients. Pathway analysis also has statistical advantages in terms of reducing the dimensionality of high-throughput datasets and provide a focused set of targets for biological validation. These properties of genetic pathway data motivated us to explore canonical correlation analysis (CCA) (Hotelling 1992) to capture the correlations between the pathways which share common genes and use this property during prediction in the machine learning framework. CCA has been explored by (Tang and Ferreira 2012) where a multivariate test of association to simultaneously test the association between a single nucleotide polymorphism (SNP) and multiple phenotypes is proposed. Lin *et al.* (2013) use CCA to study the mutual relationship between SNP and gene expression data.

**Figure 1.**
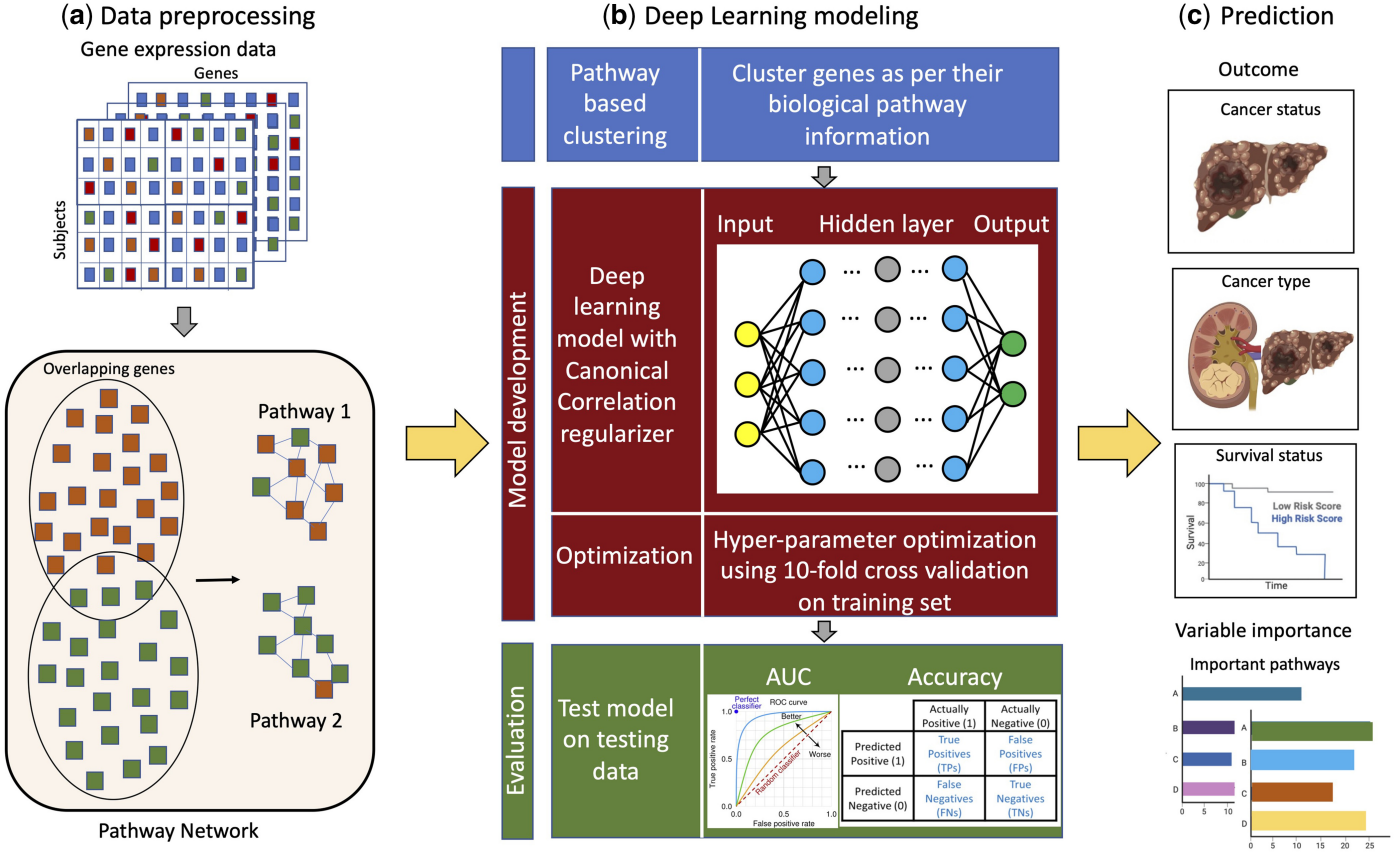
Broad overview of our objective on predicting outcomes from genetic pathway data through NN modeling. (a) Preprocess the gene expression data and group the genes based on their biological pathways; (b) provide the pathway data as input to the NN modeling with Canonical Correlation-based regularization and evaluate the performance of the model on training and test sets; (c) predicting outcomes for variety of tasks and identifying important pathways and genes relevant to the disease prediction

However, currently, as per our knowledge, there is no comprehensive deep learning methodology to efficiently assess disease prediction taking into consideration overlapping genes between pathways using a regularization approach. In this article, we propose an end-to-end framework for combining genetic data into pathway structure and using CCA along with neural network (NN) modeling for prediction of disease status and outcomes. A broad overview of our goal is illustrated in Fig. 1. We aim to explore the complex interactions within the gene sets and between pathways and also capture the correlations between pathways to understand the role of genetic data during prediction.

**Figure 2.**
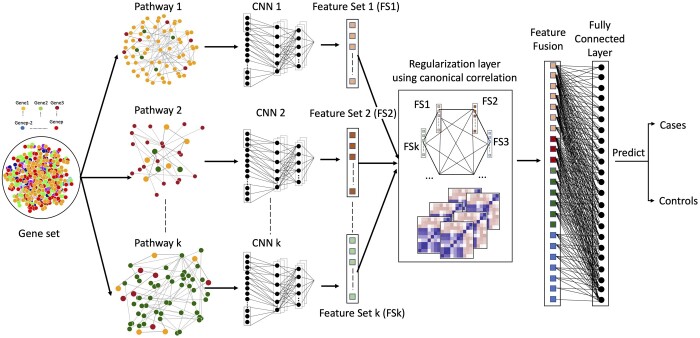
Overall framework for the ReGeNNe; (from left to right) pathway clustering from gene set; stratified CNN module for feature extraction from pathway data; Canonical Correlation-based regularization layer for fusion of individual pathway data features and binary prediction using softmax function in the fully connected layer of the NN

To take advantage of end-to-end deep learning and prediction through genetic data while collaboratively benefiting from capturing the correlations between the gene pathways, we propose a framework for an ensemble-based NN modeling *ReGeNNe* as shown in Fig. 2. We adopt a regularization approach using correlation-maximizing regularizer for fusion of features from individual pathways. We implement our proposed regularizers collectively as a correlation-regularized network layer using CCA. Our proposed framework consists of two components: (i) extracting features from the gene sets within each pathway using convolutional neural networks (CNNs) and (ii) fusion of the features obtained from individual pathways using a CCA regularization layer. Our two-step framework is empowered by the CNN’s capacity for making robust strategies to extract nonlinear features without the explicit need to manually input the complex relationships from individual pathways. Further, through CCA layer we fuse the features obtained from the ensemble of CNNs operating on the pathways for prediction tasks. We propose a computational pipeline, wherein, the input to the feature selection/extraction module is a vector representing a normalized gene set corresponding to the subjects. We preprocess the genetic data to exhibit spatial taxonomic similarity, so that the CNN can extract the gene data features efficiently from each pathway. The selected or extracted features are then passed to the CCA module (Chen and Yao 2009, Gu *et al.* 2018, Wang *et al.* 2020) for feature fusion from multiple pathways. During prediction, we also address the important issue of interpretability of NN models, where, through an integrated gradient (IG) approach (Sundararajan *et al.* 2017) we identify the pathways and genes playing an important role during prediction. We implement our methodology in three real cancer genetic datasets for different prediction tasks to show our model’s generalizability and applicability in different scenarios.

## 2 Materials and methods

### 2.1 Feature extraction from individual pathways through CNNs

In this step, firstly, the individual genes are mapped to their respective pathways and given as input to CNN models. CNNs are known to take advantage of local spatial coherence of input data in turn being able to dramatically reduce the number of operations needed to process the input due to convolution on patches of adjacent nodes using the local connectivity property. Making use of this property, within each pathway, genes are ordered based on their correlation with each other using Spearman correlation rank to make the adjacent genes in the input data similar in nature to accurately capture spatial structure during network learning. Such an ordering based on correlation/similarities between the genes is conducted to fulfill the requirement of the CNN for the input nodes to demonstrate spatial similarities (Sharma *et al.* 2020). Spearman correlation matrix for genes in the data is represented as a g×g matrix for *g* genes in a pathway. Next, this matrix is vectorized with each row represented by a single coefficient, calculated with respect to all the genes as:


(1) $$\rho_{\mathrm{gen}e_{row_{j}}}=\sqrt[{g}]{\left|\rho_{\mathrm{gen}e_{j1}}|\cdot |\rho_{\mathrm{gen}e_{j2}}|\ldots .\cdot |\rho_{\mathrm{gen}e_{jg}}\right|}$$

for j∈[1,g]

The correlation vector thus obtained is represented as ${\text{Corr}}_{\mathrm{gene}}$ as:


(2) $${\text{Corr}}_{\mathrm{gene}}=\{\rho_{\mathrm{gen}e_{row_{1}}},\rho_{\mathrm{gen}e_{row_{2}}}\ldots \;.\;.,\rho_{\mathrm{gen}e_{row_{g}}}\}$$

The values in the set ${\text{Corr}}_{\mathrm{gene}}$ (refer Equation 2) are then sorted in a decreasing order to obtain a new vector ${\text{Corr}}_{\mathrm{gene}}^{\prime }$ containing correlation coefficients in decreasing order which are further re-indexed from 1 to *g*. The ${}^{\prime }$ here represents re-indexing.


(3) $${\text{Corr}}_{\mathrm{gene}}^{\prime }=\{\rho_{\mathrm{gen}e_{\mathrm{row}5}},\rho_{\mathrm{gen}e_{\mathrm{row}3}}\ldots ..,\rho_{\mathrm{gen}e_{row_{k}}}\}$$

(4) $${\text{Corr}}_{\mathrm{gene}}^{\mathrm{sorted}}=\{\rho_{\mathrm{gen}e_{row_{1}}},\rho_{\mathrm{gen}e_{row_{2}}}\ldots ..,\rho_{\mathrm{gen}e_{row_{g}}}\}$$

s.t. $\rho_{\mathrm{gen}e_{row_{1}}}>\rho_{\mathrm{gen}e_{row_{2}}}\ldots >\rho_{\mathrm{gen}e_{row_{g}}}$

Subsequently, the heatmap obtained by the correlations in the gene data is reordered based on the decreasing order of the cumulative correlation coefficients as shown in Supplementary Fig. S2. Through this ordering the correlation structure between the genes can be used to establish a similarity in the neighboring genes before being provided to the CNN model. Further, these ordered genes in each pathway are presented as input to individual CNNs.

### 2.2 Canonical correlation regularization layer

**Figure 3.**
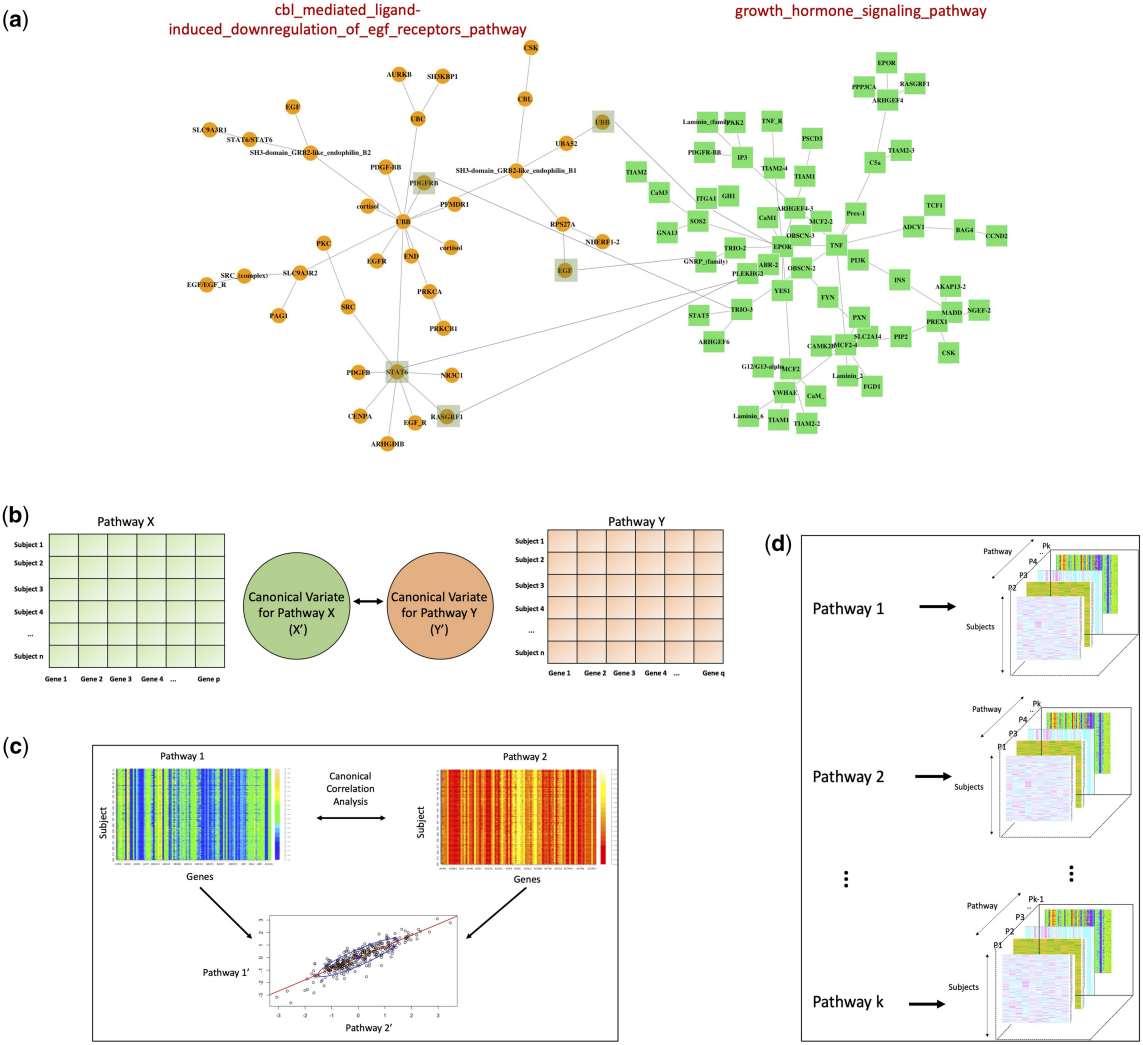
Interactions between pathways due to sharing gene sets. (a) Example illustration of two pathways from TCGA liver cancer dataset (Liu *et al.* 2018) sharing five genes in common represented by joint square and circle, (b) pathway X with “p” genes and Pathway Y with “q” genes across all subjects reduces to canonical covariates, respectively, (c) actual representation of two pathway matrices into canonical correlation variates in the 2D space from the TCGA liver cancer dataset, and (d) demonstrating the complexity of the regularization layer as all pathways pairs are considered in the interactive network. Therefore, if there are total “k” pathways, each pathway's paired interaction with k-1 rest pathways is incorporated into the CCA layer

For fusion/ensembling of the features obtained from individual pathways we incorporate a Canonical Correlation regularization layer (Chen and Yao 2009, Gu *et al.* 2018). CCA (Hotelling 1992) determines the relationship between variable sets from two domains of measurement. Given two sets X and Y of dimensions p and q, the first CCA mode is reflected in a linear combination of the variables in X and another in linear combination of the variables in Y (refer Fig. 3b and c and Supplementary Fig. S1) as:


(5) $$X^{\prime }=a^{T}x,a\in R^{p}$$

(6) $$Y^{\prime }=b^{T}y,b\in R^{q}$$

and the Canonical correlation (CanCorr) between the two sets is represented as:


(7) $$\rho =\mathrm{CanCorr}(X^{\prime },Y^{\prime })=\mathrm{Cancorr}(a^{T}x,b^{T}y)$$

To utilize CCA in our CNN network, we start with a CNN composed of L layers. Denote its network parameters as


(8) $$\Theta =W_{l},b_{l},l\in L$$

where $W_{l}$ and $b_{l}$ are respectively the weight matrix and bias vector associated with the *l*th network layer. In the setting of supervised learning, given M training samples $S=s^{i}$, *i* = 1 to *M*, the network parameters Θ are optimized by minimizing the empirical risk:


(9) $$\frac{1}{M}\sum_{i=1}^{M}Loss(s^{i};\Theta )$$

where Loss(·) is a function such as cross-entropy loss. Adding regularization to the network training objective results in the following optimization problem:


(10) $$min_{\Theta }\frac{1}{M}\sum_{i=1}^{M}Loss(s^{i};\Theta )+\lambda R(\Theta )$$

where *R*(·) is the regularizer to be specified, and λ is a trade-off parameter.

For a specified *l*th network layer, $f(z)=f(W^{T}x+b)$ where f(·) is an element-wise nonlinear activation function such as Rectified Linear Unit.

Therefore, if we have two pathway sets where $X_{1}$ is the feature vector obtained from pathway 1 and $X_{2}$ is the feature vector obtained from pathway 2, $W_{1}$ and $W_{2}$ are subsequent weight vectors. Given M training samples. $X_{1}=[x_{1}^{1},\ldots ,x_{M}^{1}]$ and $X_{2}=[x_{1}^{2},\ldots ,x_{M}^{2}]$ of training set *S*, applying CCA to the $l_{th}$ network layer amounts to optimizing $W_{1}$ and $W_{2}$ and the CCA between the two pathways is represented as:


(11) $$\mathrm{CanCorr}(X_{1},X_{2})=max_{W_{1},W_{2}}\frac{1}{M}tr(W_{1}^{T}X_{1}X_{2}^{T}W_{2})$$

s.t. $\frac{1}{M}tr(W_{1}^{T}X_{1}X_{1}^{T}W_{1})=\frac{1}{M}tr(W_{2}^{T}X_{2}X_{2}^{T}W_{2})=I$, where *I* is an identity matrix of compatible size.

Under the framework of regularized function learning, we train network parameters by penalizing objectives of the main learning tasks with CCA regularization such as:


(12) $$min_{\Theta }\frac{1}{M}\sum_{i=1}^{M}Loss(s^{i};\Theta )-\lambda \mathrm{CanCorr}(X_{1},X_{2})$$

We then forward the output of the canonical regularization layer to the final fully connected layer which has a binary node with a softmax activation (Goodfellow *et al.* 2016) function given by the equation:


(13) $$\sigma {\left(z\right)}_{j}=\frac{exp^{z_{j}}}{\sum_{k=1}^{K}exp^{z_{k}}}$$

for *j* = 1, …, *K*. We can see that the softmax function normalizes a K dimensional vector *z* of arbitrary real values into a *K* dimensional vector σ(z) whose components sum to 1. The loss function that is minimized on softmax output layer equipped neural nets is the cross-entropy loss:


(14) $$H(m,n)=-\sum_{i}m_{i}(\text{log}(n_{i}))=-y\;\text{log}\;\hat{y}-(1-y)\;\text{log}(1-\hat{y})$$

Assuming, *m* and *n* are discrete distributions, where *y* is the true label for some iteration *i* and $\hat{y}$ is the NN output at iteration *i* predicting the binary disease status.

### 2.3 Extract important features through NNs

For identifying the important features in our predictive modeling, we use the IG methodology which is an interpretability technique for deep NNs (Sundararajan *et al.* 2017). We calculate gradients to measure the relationship between changes to a feature and corresponding changes in the model’s predictions. Formally, suppose we have a function $F:R_{n}\Rightarrow [0,1]$ that represents a deep network. Let $x\in R_{n}$ be the input at hand, and $x\in R_{n}^{\prime }$ be the baseline input (zero embedding vector). We consider the interpolation from the baseline *x*′ to the input *x*, and compute the gradients at all points along the path. IGs are defined as the path integral of the gradients along the straightline path from the baseline *x*′ to the input *x*. The gradient informs which feature has the strongest effect on the models predicted class probabilities. Varying the input variable changes the output, and hence each input feature will receive some attribution during this interpolation which in turn helps to calculate the feature importance for the input. The higher the gradient, more important the feature is.

## 3 Results

### 3.1 Data description

Given the remarkable advances in high-throughput technologies, the development of The Cancer Genome Atlas (TCGA) (Liu *et al.* 2018) database provides an abundance of high-quality information regarding various cancer datasets. In this manuscript, we conducted our analysis on three real cancer genetic datasets. Gene expression and pathway data for the cancer patients were obtained from the TCGA dataset of the Broad Institute GDAC Firehose (Liu *et al.* 2018). Pathway data was obtained through the PARADIGM algorithm which integrates pathway, expression and copy number data to infer activation of pathway features within a superimposed pathway (SuperPathway) network structure. The SuperPathway structure comprises of 1500 constituent pathways from three pathway databases, NCI-PID, BioCarta and Reactome, containing 19 000 pathway features, representing 7369 genes, 9354 complexes, 2092 families, 82 RNAs, 15 miRNAs, and 592 abstract processes. This dataset contains the PARADIGM integrated pathway levels (IPLs) of 19K pathway features of whitelisted Pan-Cancer 33 samples from this cohort, computed using the platform corrected RNA-seq and GISTIC thresholded gene level copy number data. The gene expression profile was measured experimentally using the Illumina HiSeq 2000 RNA Sequencing platform by the University of North Carolina TCGA genome characterization center. Level 3 data was downloaded from TCGA data coordination center. This dataset shows the gene-level transcription estimates, as in log2(*x* + 1) transformed RSEM normalized count. Genes are mapped into the human genome coordinates using UCSC Xena HUGO probeMap (see ID/Gene mapping link below for details). All the three datasets comprised of 19 503 genes and 1387 pathways.

**Kidney cancer dataset**: The outcome space consisted of 945 subjects with 553 subjects with early-stage kidney cancer (Stage I and II) and 392 with late-stage kidney cancer (Stage III and IV). Our algorithm aimed at classifying early or late-stage kidney cancer based on their genetic and pathway information.

**Combined liver and kidney cancer dataset**: In this dataset, we aimed at differentiating between the cancer type (liver/kidney) based on the genetic data. We combined the samples from the liver and kidney cancer dataset to create a combined cohort of subjects. In this dataset, we had a total of 1383 subjects with 945 subjects for kidney cancer and 438 for liver cancer.

**Ovarian cancer dataset**: In this study, the outcome space comprised of 362 subjects with overall survival at 2 years. We split the patients into alive and deceased groups with respect to their survival status. Patients whose survival days were <2 years were removed from our subject population. 120 subjects were alive at 2 years and 242 were categorized as dead based on the 2-year survival status.

### 3.2 Experimental setting

For the NN modeling, the datasets were divided into 70% as the training data to train the *ReGeNNe* model and 30% as the test data to validate the model performance. An internal validation was done using 10 times 10-fold cross validation on the training set, to analyze model performance before testing and to eliminate overfitting. For each fold of the cross-validation, 90% of the total training set was selected at random for training, and the remaining 10% was selected as a holdout set for testing. We obtained 10 Area Under the Curve (AUC) values corresponding to initial 10-folds in the training set. We repeated this process 10 times in order to generate corresponding 100 AUC values. We then calculated the 95% confidence intervals (CIs) using these 100 AUC values. Our CNN framework is presented in Supplementary Fig. S8. We defined the model with two 1D CNN layers, each followed by a pooling layer. The pooling layer reduces the learned features to half their size, consolidating them to only the most essential elements. Input signifies the dimension of the input to the layer. We finally obtain the flattened feature vectors corresponding to each pathway and further send it to the CCA module for feature fusion.

Four-hundred epochs were run for the NN model with a stride size of 1, batch size of 5 and number of filters in the CNN network as 32 (refer Supplementary Table S2). Each network was trained using stochastic gradient descent with a learning rate of 0.001. We trained our network on an NVIDIA Tesla P100 GPU with 16 GB of RAM using tensorflow library in Python along with some data analysis using R version 4.3.0. The performance of our technique was evaluated through mean Area Under the Receiver Operating Characteristics Curve (AUC) and the 95% CI of the AUC values. To reduce the dimensionality of pathway data we screen the important pathways that make a significant contribution toward disease prediction, through the CNN network by generating AUCs for all pathways individually and sorting the AUCs in the decreasing order. The top 100 pathways contributing to the highest performance in disease prediction for each of the cancer outcome were selected for the Canonical Correlation Regularization-based fusion.

For CCA, as shown in Fig. 3a, we observe that the two pathways share common genes in TCGA liver cancer dataset (Liu *et al.* 2018). Hence, CCA can be utilized to capture this association in an accurate manner as shown in Fig. 3b and c. We would also like to emphasize on the complexity of the fusion task at hand, i.e. if we have *k* pathways in the dataset, each pathway is leading to k-1 interactions with other pathways in the dataset as shown in Fig. 3d.This fusion in the CCA layer makes the CCA computation intricate and complex and thus, justifies the utilization of automated CCA regularization layer.

For a fair comparison, we directly compared the ReGeNNe model which is essentially a stratified CNN with regularization with a stratified CNN without regularization to see the effectiveness of the CCA regularization layer. Further, we also compared the results obtained by our proposed model against conventional machine learning models which includes, Random Forests (RF) (Liaw *et al.* 2002), Ridge regression (Hoerl and Kennard 1970), Lasso regression (Tibshirani 1996), and three deep learning models, stratified CNN without regularization (Sharma *et al.* 2020), Fully Connected Neural Network (Zhang *et al.* 2017b) and basic CNN (Krizhevsky *et al.* 2012). We ensured hyperparameter tuning of all our ML models as shown in Supplementary Tables S3 and S4 to select the best model for evaluation. The Type 1 error performance where we tested for our *ReGeNNe* model under the null is shown in Supplementary Table S1.

### 3.3 Evaluation of predictive performance

#### 3.3.1 10-Fold cross validation on the training set

**Figure 4.**
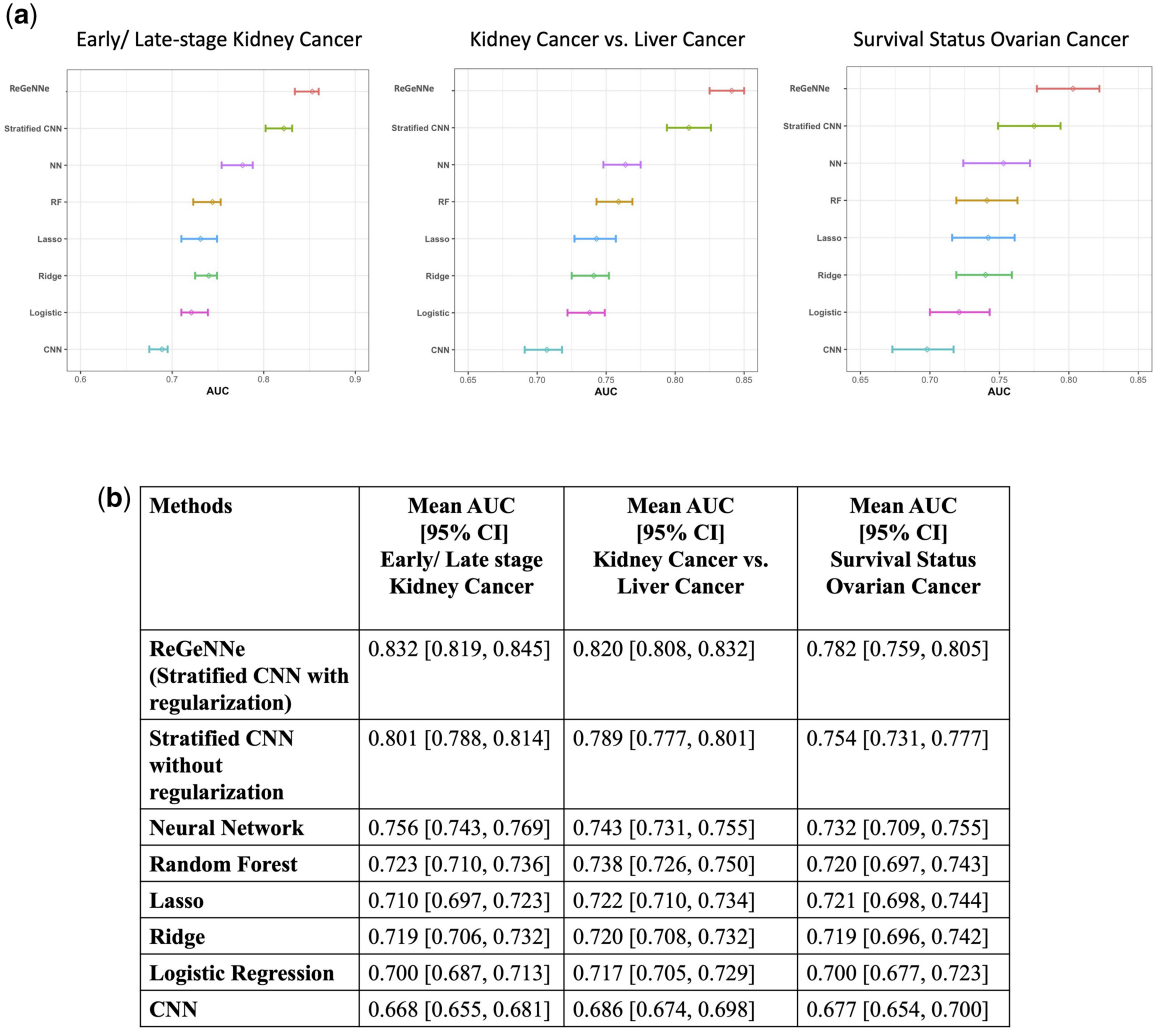
Performance evaluation. (a) 95% CIs obtained for the mean AUC values for 10 times 10-fold cross validation on the training set for predicting early/late-stage kidney cancer, distinguishing between kidney versus liver cancer and predicting survival status in ovarian cancer; (b) performance on the test sets of the three datasets in terms of mean AUC and 95% CIs of the prediction models. First row shows consistent improvement and higher accuracy as observed through our proposed modeling on the three datasets

For the three datasets we evaluated the 10-fold cross validation performances on their training set as shown in Fig. 4a. We observed that our proposed model *ReGeNNe* consistently performed better in terms of mean AUC and 95% CI as compared to other conventional ML approaches. Stratified CNN without considering interaction between pathways through regularization was next in performance to our methodology with mean AUC of 0.822, 0.775, and 0.81 on the kidney cancer, kidney versus liver cancer and ovarian cancer datasets respectively showing that stratification based on pathway structure aids in improving predictive ability as compared to other approaches. Incorporating the interaction between pathways through our approach further improves mean AUC to 0.853, 0.803, and 0.841 on the kidney cancer, kidney versus liver cancer, and ovarian cancer datasets.

#### 3.3.2 Results on the test set

**Predicting early- and late-stage kidney cancer**: We conducted the validation analyses on the test set of the Kidney cancer dataset (tabulated in Fig. 4b Column 2) and calculated CIs using bootstrapping (500 times) on the test data. In this dataset, the outcome was predicting early-stage and later-stage kidney cancer. We observed that our proposed CCA regularized CNN approach outperformed all the other machine learning and statistical approaches in terms of the AUC values with AUC 0.832 [95% CI: 0.845, 0.818]. The approach of the stratified CNN framework without using regularization gave an AUC of 0.801 [95% CI: 0.788, 0.814]. We observed that CNN framework taking input without any adjacent similarity performed the lowest due to requirements of CNN network not satisfied. Fully connected NNs performed well with AUC of 0.756 [95% CI: 0.743, 0.769], capturing the nonlinear relationships between the genes. Moreover, amongst the conventional models, RF due to its tree structure capable of understanding the nonlinear nature and complex interactions performed best amongst the conventional models with and AUC of 0.723 [95% CI: 0.710, 0.736].

**Predicting type of cancer, liver, or kidney**: We also tested our modeling in distinguishing between cancer types namely liver and kidney in subjects based on their genetic data. As tabulated in Fig. 4b Column 3, we observed that our methodology *ReGeNNe* consistently performed better in terms of mean AUC and CIs of 0.820 [95% CI: 0.808, 0.832] followed by stratified CNN 0.789 [95% CI: 0.777, 0.801] and NN methodology 0.743 [95% CI: 0.731, 0.755]. Amongst the conventional models RF gave an AUC of 0.738 [95% CI: 0.726, 0.750], lasso and ridge regression performed closely with mean AUCs of 0.722 [95% CI: 0.710, 0.734] and 0.720 [95% CI: 0.708, 0.732] respectively followed by logistic Regression 0.717 [95% CI: 0.705, 0.729] and CNN framework 0.686 [95% CI: 0.674, 0.698].

**Predicting 2-year survival outcome in ovarian cancer**: While predicting the survival rate in subjects with Ovarian Cancer, our method performed the best with an AUC of 0.782 [95% CI: 0.759, 0.805] (refer Column 4, Fig. 4b). The AUC value here was observed to be lower than the one observed with Kidney Cancer due to less number of subjects for the prediction task. The performance was followed by Stratified CNN and NNs with AUCs 0.754 [95% CI: 0.731, 0.777] and 0.732 [95% CI: 0.709, 0.755] respectively showing that deep learning models are able to identify the relationship between gene data and outcome in a better manner as compared to conventional models such as RF (AUC = 0.720 [95% CI: 0.697, 0.743]) and Lasso and Ridge Regression (AUC = 0.721 [95% CI: 0.698, 0.744] and 0.719 [95% CI: 0.696, 0.742] respectively). ROC curves on these datasets are provided in Supplementary Figs S3–S5.

**Figure 5.**
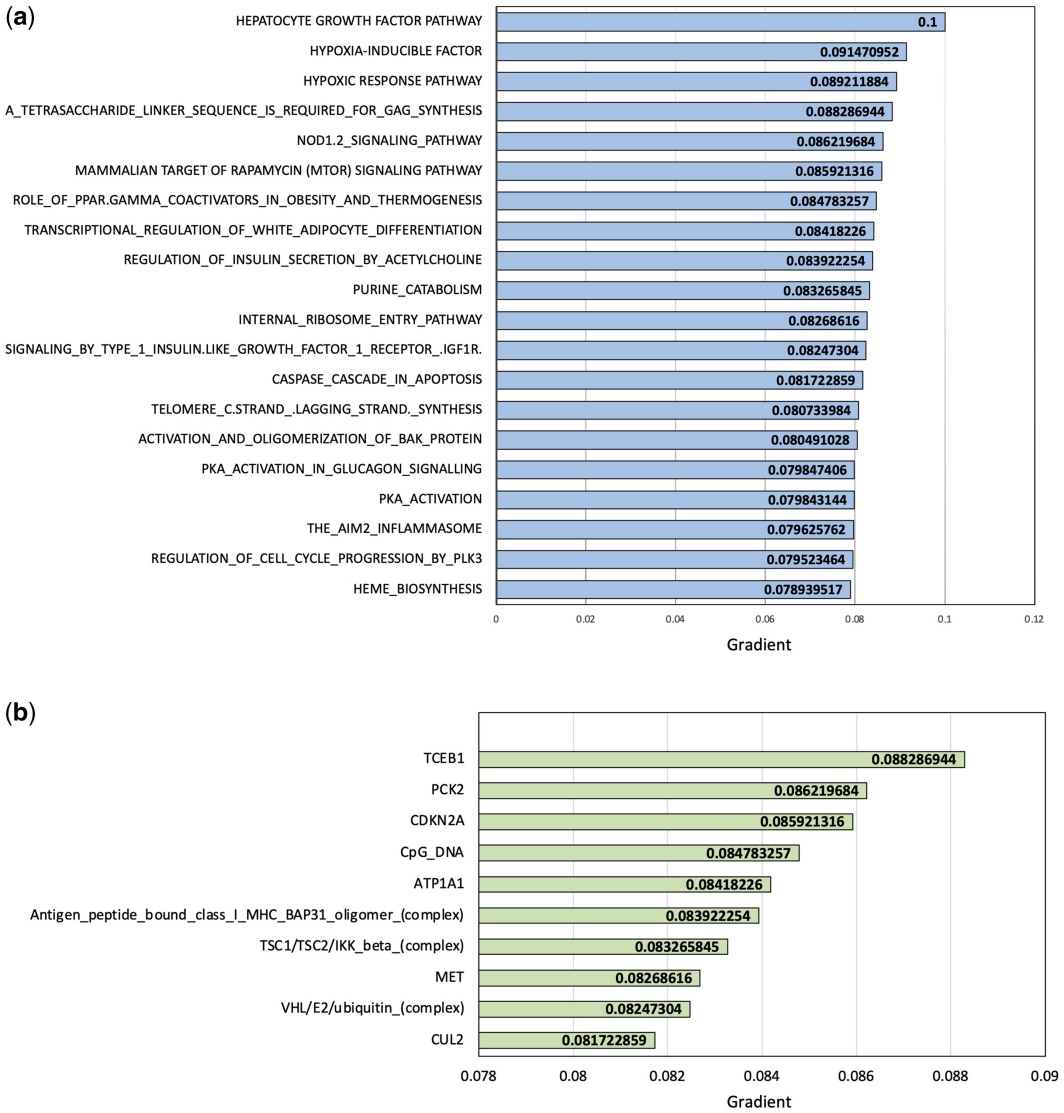
Identifying variable importance through IG approach in NN modeling. (a) Top-20 important pathways for kidney cancer stage prediction and (b) top-10 important genes for kidney cancer stage prediction. The higher the gradient more important the feature

**Important pathways and important genes**: Through the IG approach (Sundararajan *et al.* 2017) (refer Section 2.3) we also identified important pathways and genes in each of the cancer types. As an example in Fig. 5a and b, we plot the top-20 ranked pathway and top-10 genes identified by our deep learning methodology as the most important while predicting early/late-stage kidney cancer. Higher the gradient more important the feature is, therefore, Hepatocyte growth factor pathway, hypoxia inducible factor, hypoxic response pathway along with TCEB1 gene, PCK2 gene and CDKN2A gene known for their existing associations with kidney cancer emerged important during our analysis showing our model’s effectiveness. For the liver versus kidney cancer and the ovarian cancer datasets their top ranked pathways are provided in Supplementary Figs S6 and S7, respectively.

## 4 Discussion

In this article, we propose *ReGeNNe*, an end-to-end deep learning framework incorporating the biological clustering of genes through pathways and further capturing the interactions between pathways sharing common genes through CCA. As discussed in Section 1, in the existing studies, genes are used for disease prediction without acknowledging the fact that genes are functioning by coordinately interacting with each other. This in turn, misses out on capturing the genetic heterogeneity across patients and dysregulation at the pathway level instead of the gene level. In addition to combining signals from a number of genes in a pathway, results from pathway analysis also can shed light on the biological processes underlying a disease. Our Canonical Correlation-based NN modeling captures linear/nonlinear dependencies between pathways, projects the features from genetic pathways into the kernel space, and ultimately fuses them together in an efficient manner for disease prediction as shown in Fig. 4. Apart from our model, we also observed that amongst the deep learning models, stratified CNN without regularization performed better than basic CNN and fully connected NNs due to its functioning on pathway level instead of gene level and thus, being able to extract better features from similar genes in a pathway. Amongst the conventional ML models, RF performed better than other approaches due to its capability of handling nonlinear dependencies well owing to its tree-based learning model.

NNs are usually perceived as black-boxes, however through applying IG methodology in our framework, we were able to identify the most important pathways and genes contributing to the prediction. This approach makes our prediction task more interpretable and takes the framework a step closer toward explainable Artificial Intelligence. Cancer is a complex biological process that involves genetic and epigenetic alterations. There has been tremendous progress in our understanding of the critical oncogenic and tumor suppressor pathways involved in cancer types (Takahashi and Nishioka 2011, Lee *et al.* 2014, Chuma *et al.* 2015, Dimri *et al.* 2017, Elattar *et al.* 2018). In the Kidney Cancer prediction task, our top identified pathways consisted of Hypoxia-Inducible Factor (HIF2), hypoxic response pathway and mammalian target of rapamycin (mTOR) signaling pathway which are validated through existing studies for their association to Kidney cancer (Hanna *et al.* 2008, Schödel *et al.* 2016, Chen *et al.* 2020). In the prediction task differentiating Kidney and Liver cancer in patients using genetic data, Receptor tyrosine pathways, phosphatidylinositol 3-kinase (PI3K), mammalian target of rapamycin (mTOR), Ras mitogen-activated protein kinase (Ras/Raf/MAPK), Wnt/β-catenin, and Hippo signaling pathways emerged as important pathways as shown in Supplementary Fig. S6. These pathways have been researched in the past and show association with liver as well as kidney cancer (Dimri and Satyanarayana 2020). In the ovarian cancer survival prediction, our methodology identified Epidermal Growth Factor receptor signaling pathway and the Activation of AKT as the top pathways known for their association to ovarian cancer (Smolle *et al.* 2013) (refer to Supplementary Fig. S7). Therefore, the interpretability of our modeling can be justified as biologically relevant to efficiently identifying pathways that play an important role in prediction tasks and can shed light on understanding the nonlinear relationships captured by the NN model. Similarly, at the gene level our modeling is able to dive deeper into the complex relationships shared between genes and outcome prediction and is able to identify TCEB1 (Hakimi *et al.* 2015), PCK2 (Chen *et al.* 2020), ATP1A1 (Zhang *et al.* 2017a) as the most important genes to kidney cancer stage prediction as shown in Fig. 5 and whose hotspot mutations are also validated by existing research (Linehan and Ricketts 2019) to have been associated to kidney cancer prognosis.

We would also like to list certain limitations of our study. For reducing the complexity in the ensembling of individual CNNs in the regularized layer and capturing the important pathways and genes while prediction, we selected top 100 pathways in our CCA regularized model (refer Section 3.2). In the future, we would develop a scalable model taking into consideration all pathways to evaluate the predictive ability in a more comprehensive manner. Our current approach was also limited to a fixed outcome definition such as, case versus control, binary survival status, etc. at single timepoint. In the future, it would be interesting to analyze our approach on datasets with varying longitudinal outcomes at different time-points, and time to event outcomes. Adding environmental factors along with gene data as an input to the NN modeling also serves as an interesting area for future research. Lastly, although our implementation on three cancer datasets shows our model’s robustness, in the future, we would explore other disease datasets to compare the predictive performance.

In conclusion, the strength of our methodology lies in efficient feature extraction from complex genetic pathway data through a CCA regularized CNN modeling. We believe pathway-based approaches that model joint effects of genetic variations in multiple functionally related genes across different pathways, are a major step forward in improving the performance of genetic data-based prediction and understanding molecular mechanisms of diseases. Despite the challenges associated with NN pathway-based analysis of genetic data, we estimate that the predictive ability and future potentials of these methods will increase as the coverage and quality of gene annotation databases improve.

## Supplementary Material

btad679_Supplementary_Data
