# Supplementary material for: ReGeNNe: genetic pathway-based deep neural network using canonical correlation regularizer for disease prediction
Source: Bioinformatics. 2023 Nov 14;39(11):btad679. doi: 10.1093/bioinformatics/btad679 (PMC10666205; doi:10.1093/bioinformatics/btad679)
Supplement: btad679_Supplementary_Data [file btad679_supplementary_data.pdf]

Supplementary Material for ‘ReGeNNe: Genetic pathway-based deep neural network using canonical correlation regularizer for disease prediction’

Contents

1 Supplementary Tables 3

2 Supplementary Figures 9

3 References 18

List of Supplementary Tables

1 Type 1 error performance of the proposed method as well as the conventional machine learning methods used for comparative analysis. 3

2 Results evaluating performance of our model by changing network parameters on the Kidney Cancer dataset [1]. The boldfaced attributes represent the parameter values for which the model performs the best. 4

3 Hyperparameters and their search range and tuned values for ReGeNNe, Stratified CNN without regularization and CNN) 5

4 Hyperparameters and their search range and tuned values for the conventional Machine Learning models 6

5 Predictive performance comparison of existing ML approaches on the three cancer cohorts using only gene expression data without considering pathway structure to ReGeNNe model 7

6 Predictive performance of ML approaches on the simulated gene expression data for different cancer cohorts 8

List of Supplementary Figures

1 Example showing a 3D-plot representing similarity between seven genes common between two pathways in the Kidney Cancer dataset captured through first three components of the Canonical Correlation Analysis 10

2 Illustration of a heatmap showing Spearman correlation coefficients of 106 genes in the pathway 1 in the Kidney Cancer dataset [1], (a) before ordering and (b) after the ordering based on correlation of the genes in the pathway 11

3 ROC curve obtained on the Kidney Cancer dataset [1] to distinguish between early and late stage cancer. The test set comprised of 165 subjects from early stage cancer category and 117 subjects from late stage cancer category. 12

4 ROC curve obtained on the Cancer dataset distinguising between liver and kidney cancer using genetic data [1]. The test set comprised of 283 subjects from kidney cancer and 131 subjects from liver cancer. 13

5 ROC curve obtained on the Ovarian cancer dataset [1] to distinguish between 2-year survivors and non-survivors. The test set comprised of 36 subjects who were alive at 2 years and 72 subjects who were not alive at 2 years. 14

6 Identifying variable importance through Integrated Gradient approach in Neural Network modeling. Top-20 important pathways distinguishing between liver and kidney cancer. 15

7 Identifying variable importance through Integrated Gradient approach in Neural Network modeling. Top-20 important pathways distinguishing between 2-year survivors and non-survivors. 16

8 Illustration of the layers in the CNN framework. (a) Framework of CNN acting on all the pathways in the input genetic data. (b) Functional working of the layers of stratified CNN on k pathways of an example dataset containing ‘p’, ‘q’, ‘r’ .... ‘s’ genes in the respective pathways. 17

1 Supplementary Tables

Type 1 error performance: To estimate the sampling distribution under the null hypothesis we generated samples with 500 permutations of our real data. For example, on predicting the stage of kidney cancer in Kidney Cancer dataset, we obtained an AUC value of 0.503 using $ReGeNNe$ and 0.501 with stratified CNN without regularization model. Comparing the AUC values obtained from our model with Neural Network (AUC=0.502), RF (AUC=0.501), Ridge (AUC=0.504), Lasso (AUC=0.503), Logistic Regression (AUC=0.502) and CNN_basic (AUC=0.501) we observed that the $ReGeNNe$ model shows no inflated type 1 error. Comparison on liver vs. kidney cancer and ovarian cancer datasets are provided in Supplementary Table 1.

Supplementary Table 1: Type 1 error performance of the proposed method as well as the conventional machine learning methods used for comparative analysis.

**Methods**	**Type I error assessment (500 permutations)**		
	**Mean AUC [95% CI] Early/ Late stage Kidney Cancer**	**Mean AUC [95% CI] Kidney Cancer vs. Liver Cancer**	**Mean AUC [95% CI] Survival Status Ovarian Cancer**
**ReGeNNe**	0.503 [0.498, 0.507]	0.501 [0.496, 0.507]	0.502 [0.496, 0.507]
**Stratified CNN without regularization**	0.501 [0.491, 0.508]	0.500 [0.491, 0.508]	0.503 [0.491, 0.508]
**Neural Network**	0.502 [0.490, 0.509]	0.502 [0.490, 0.509]	0.502 [0.490, 0.509]
**Random Forest**	0.501 [0.491, 0.507]	0.501 [0.491, 0.507]	0.501 [0.491, 0.509]
**Lasso**	0.503 [0.492, 0.510]	0.503 [0.492, 0.510]	0.504 [0.492, 0.510]
**Ridge**	0.504 [0.495, 0.511]	0.504 [0.495, 0.511]	0.502 [0.495, 0.510]
**Logistic Regression**	0.502 [0.492, 0.510]	0.502 [0.492, 0.510]	0.501 [0.492, 0.509]
**CNN**	0.502 [0.491, 0.513]	0.502 [0.491, 0.513]	0.500 [0.491, 0.511]

Supplementary Table 2: Results evaluating performance of our model by changing network parameters on the Kidney Cancer dataset [1]. The boldfaced attributes represent the parameter values for which the model performs the best.

Performance Analysis		
Parameter	Values	AUC
Stride Size	**1**	**0.832**
	2	0.821
	3	0.811
	4	0.807
	5	0.805
Window Size	3	0.822
	4	0.817
	**5**	**0.835**
	6	0.819
	7	0.818
No. of filters	16	0.819
	**32**	**0.831**
	64	0.808

Supplementary Table 3: Hyperparameters and their search range and tuned values for ReGeNNe, Stratified CNN without regularization and CNN)

**Hyperparameter**	**Value**	**Search Range**
Kernel size for Convolution	20	3, 7, 15, 20, 30, 40, 50
Pooling method	Max pooling	Max pooling, Average pooling
Number of units in hidden layer	400	100-600
Feature Scaling	Standard	Min-Max and Standard scaler
Number of Layers	3	1,3,5,7
Hidden units/ layer<br>Number of epochs	32<br>400	12,24,32,48,64,96,192<br>100-600
Dropout rate	0.15	[0,0.8]
Learning Rate	0.005	[0.0001,0.1]
Optimization solver	Adam	Stochastic Gradient Descent, Adam

Supplementary Table 4: Hyperparameters and their search range and tuned values for the conventional Machine Learning models

**Approaches**	**Parameter**	**Value**	**Search Range**
Neural Network	Learning Rate	0.005	[0.0001,0.1]
	Optimization solver	Adam	Stochastic Gradient Descent, Ad
	Feature Scaling	Standard	Min-Max and Standard scaler
	Number of Layers	3	1,3,5,7
	Hidden units/ layer	24	12,24,48,96,192
	Number of epochs	400	100-600
	Dropout rate	0.15	[0,0.8]
RF	No. of Trees	400	[10, 500]
	Mtry (No. of columns to randomly select at each level)	380	[50,400]
SVM Classifier	C	1	[0.1, 100]<br>'linear', 'poly', 'rbf', 'sigmoid'
	Kernel	sigmoid	'linear', 'poly', 'rbf', 'sigmoid'
Lasso Regression	alpha	0.005	[0.001, 0.1]
Ridge Regression	alpha	0.005	[0.001, 0.1]
Logistic Regression	C	0.001	[0.1,100]

Supplementary Table 5: Predictive performance comparison of existing ML approaches on the three cancer cohorts using only gene expression data without considering pathway structure to ReGeNNe model

	Methods	Mean AUC [95% CI] Early/ Late stage Kidney Cancer	Mean AUC [95% CI] Kidney Cancer vs. Liver Cancer	Mean AUC [95% CI] Survival Status Ovarian Cancer
Only gene expression data	CNN	0.669 [0.653, 0.680]	0.682 [0.672, 0.695]	0.675 [0.650, 0.691]
	Neural Network	0.709 [0.695, 0.720]	0.721 [0.708, 0.730]	0.715 [0.695, 0.740]
	Random Forest	0.720 [0.709, 0.729]	0.718 [0.707, 0.728]	0.713 [0.690, 0.739]
	Lasso	0.701 [0.685, 0.712]	0.715 [0.705, 0.725]	0.701 [0.675, 0.720]
	Ridge	0.751 [0.740, 0.765]	0.742 [0.729, 0.750]	0.730 [0.705, 0.749]
	Logistic Regression	0.724 [0.709, 0.737]	0.733 [0.722, 0.745]	0.715 [0.695, 0.739]
Gene Expression data + Pathway Structure	ReGeNNe	**0.832 [0.819, 0.845]**	**0.820 [0.808, 0.832]**	**0.782 [0.759, 0.805]**

Supplementary Table 6: Predictive performance of ML approaches on the simulated gene expression data for different cancer cohorts

**Methods**	**Mean AUC [95% CI] Early/ Late stage Kidney Cancer**	**Mean AUC [95% CI] Kidney Cancer vs. Liver Cancer**	**Mean AUC [95% CI] Survival Status Ovarian Cancer**
ReGeNNe	0.823 [0.808, 0.836]	0.811 [0.797, 0.823]	0.773 [0.748, 0.796]
Stratified CNN without regularization	0.792 [0.777, 0.805]	0.78 [0.766, 0.792]	0.745 [0.72, 0.768]
Neural Network	0.747 [0.732, 0.761]	0.734 [0.72, 0.746]	0.723 [0.698, 0.746]
Random Forest	0.714 [0.699, 0.727]	0.729 [0.715, 0.741]	0.711 [0.686, 0.734]
Lasso	0.701 [0.686, 0.714]	0.713 [0.699, 0.725]	0.712 [0.687, 0.735]
Ridge	0.711 [0.695, 0.723]	0.711 [0.697, 0.723]	0.71 [0.685, 0.733]
Logistic Regression	0.691 [0.676, 0.704]	0.708 [0.694, 0.72]	0.691 [0.666, 0.714]
CNN	0.659 [0.644, 0.672]	0.677 [0.663, 0.689]	0.668 [0.643, 0.691]

2 Supplementary Figures

Supplementary Figure 1: Example showing a 3D-plot representing similarity between seven genes common between two pathways in the Kidney Cancer dataset captured through first three components of the Canonical Correlation Analysis

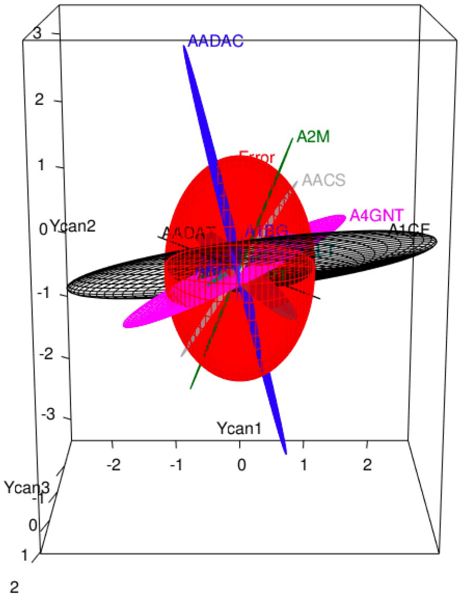

Supplementary Figure 2: Illustration of a heatmap showing Spearman correlation coefficients of 106 genes in the pathway 1 in the Kidney Cancer dataset [1], (a) before ordering and (b) after the ordering based on correlation of the genes in the pathway

(a)

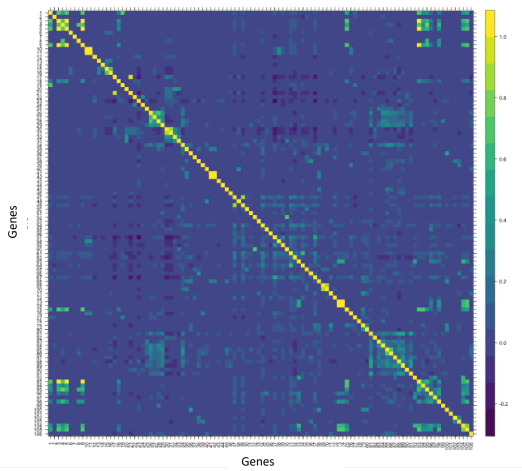

(b)

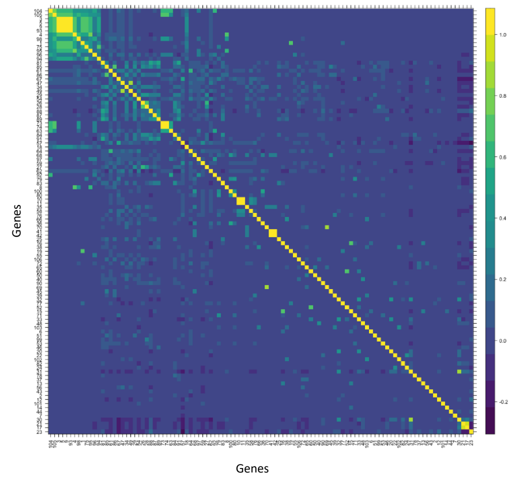

Supplementary Figure 3: ROC curve obtained on the Kidney Cancer dataset [1] to distinguish between early and late stage cancer. The test set comprised of 165 subjects from early stage cancer category and 117 subjects from late stage cancer category.

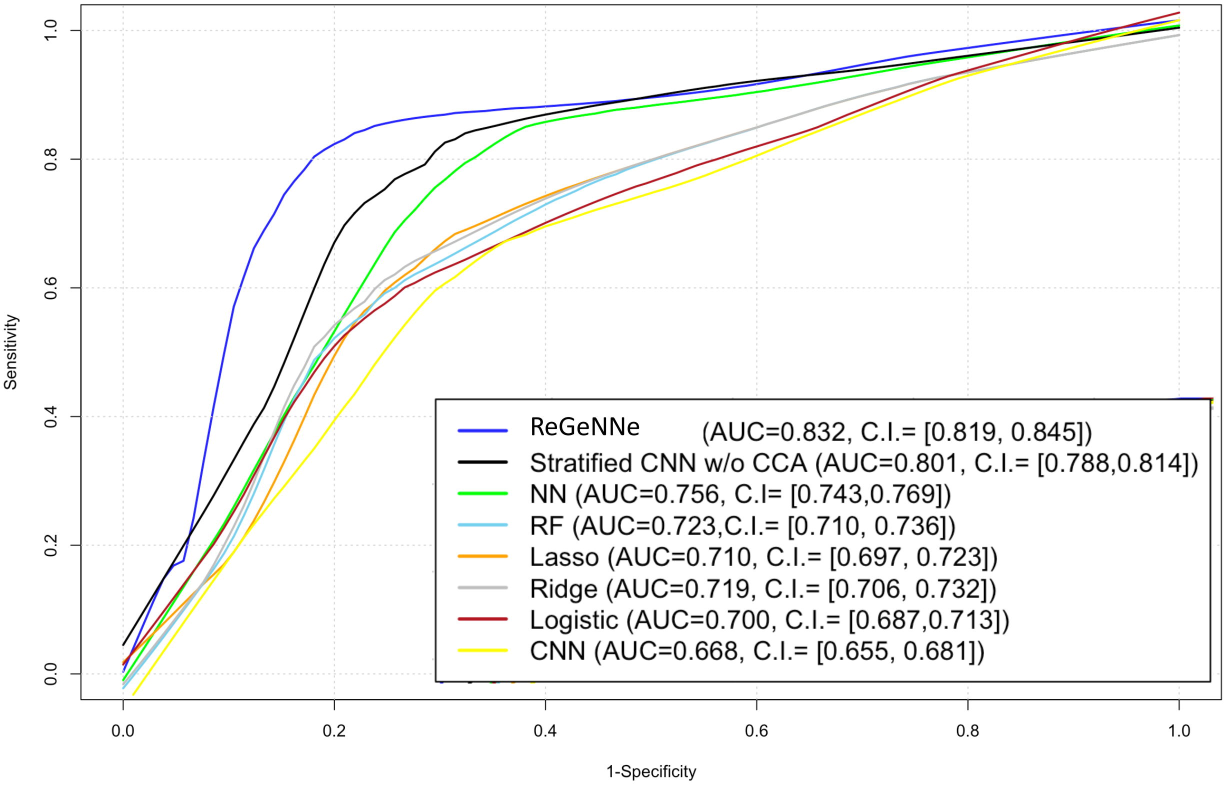

Supplementary Figure 4: ROC curve obtained on the Cancer dataset distinguising between liver and kidney cancer using genetic data [1]. The test set comprised of 283 subjects from kidney cancer and 131 subjects from liver cancer.

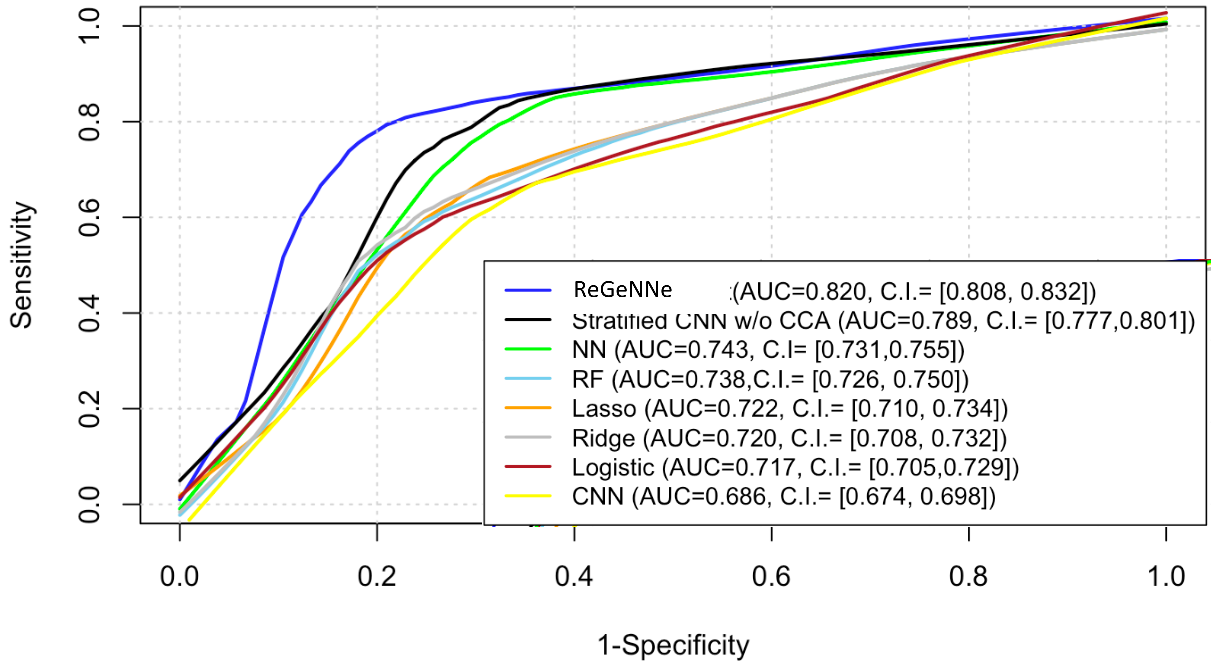

Supplementary Figure 5: ROC curve obtained on the Ovarian cancer dataset [1] to distinguish between 2-year survivors and non-survivors. The test set comprised of 36 subjects who were alive at 2 years and 72 subjects who were not alive at 2 years.

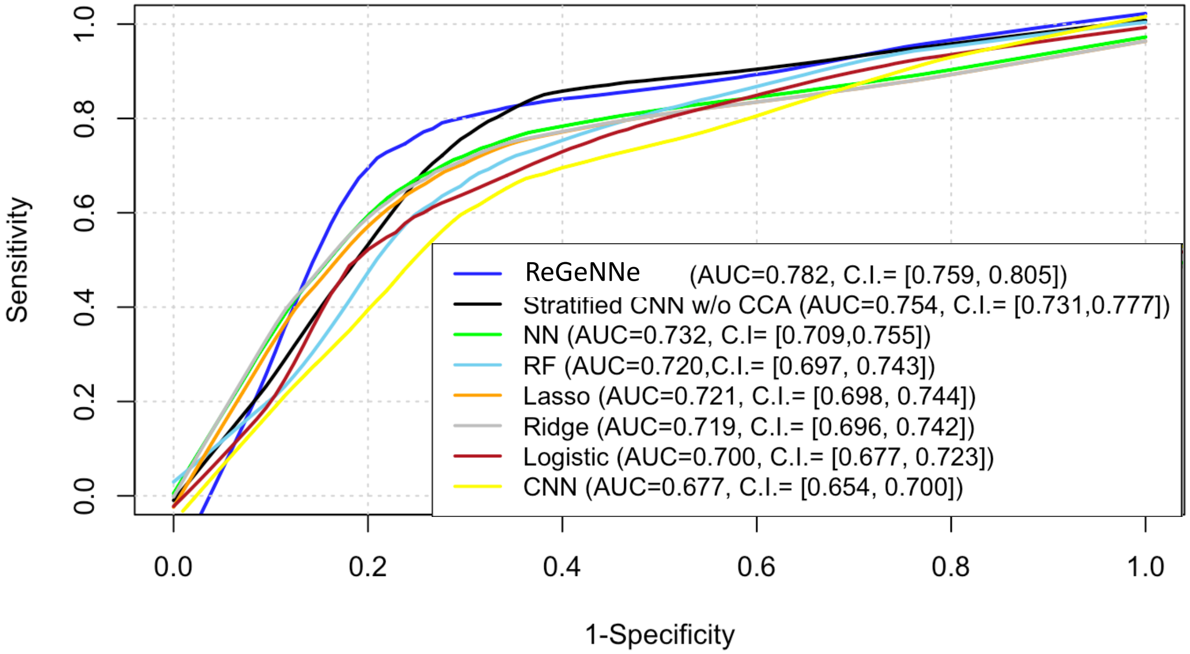

Supplementary Figure 6: Identifying variable importance through Integrated Gradient approach in Neural Network modeling. Top-20 important pathways distinguishing between liver and kidney cancer.

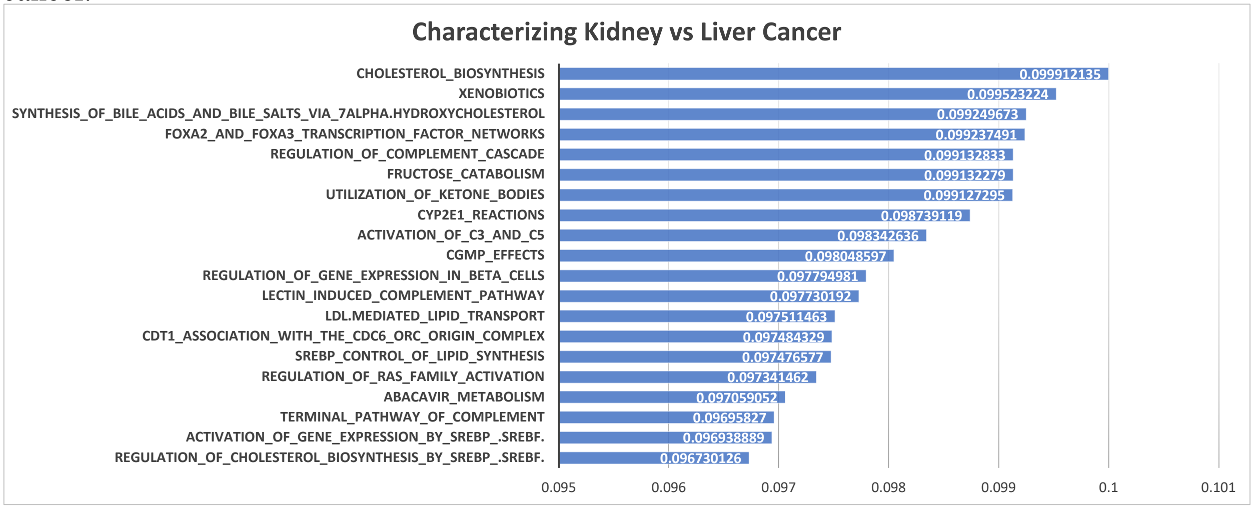

Supplementary Figure 7: Identifying variable importance through Integrated Gradient approach in Neural Network modeling. Top-20 important pathways distinguishing between 2-year survivors and non-survivors.

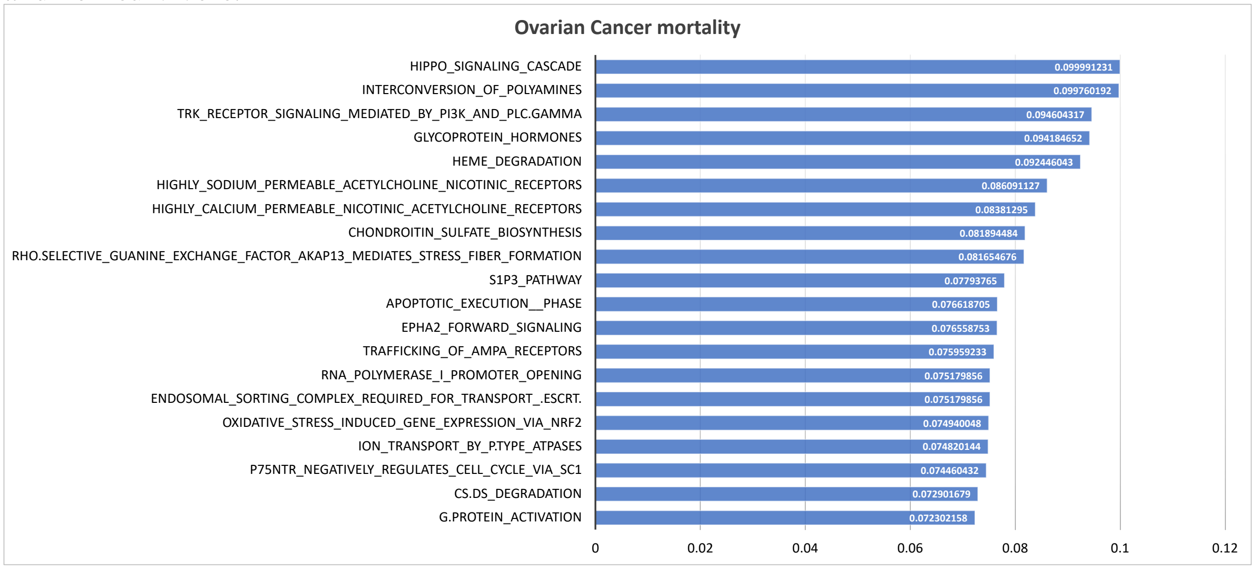

Supplementary Figure 8: Illustration of the layers in the CNN framework. (a) Framework of CNN acting on all the pathways in the input genetic data. (b) Functional working of the layers of stratified CNN on k pathways of an example dataset containing ‘p’, ‘q’, ‘r’ .... ‘s’ genes in the respective pathways.

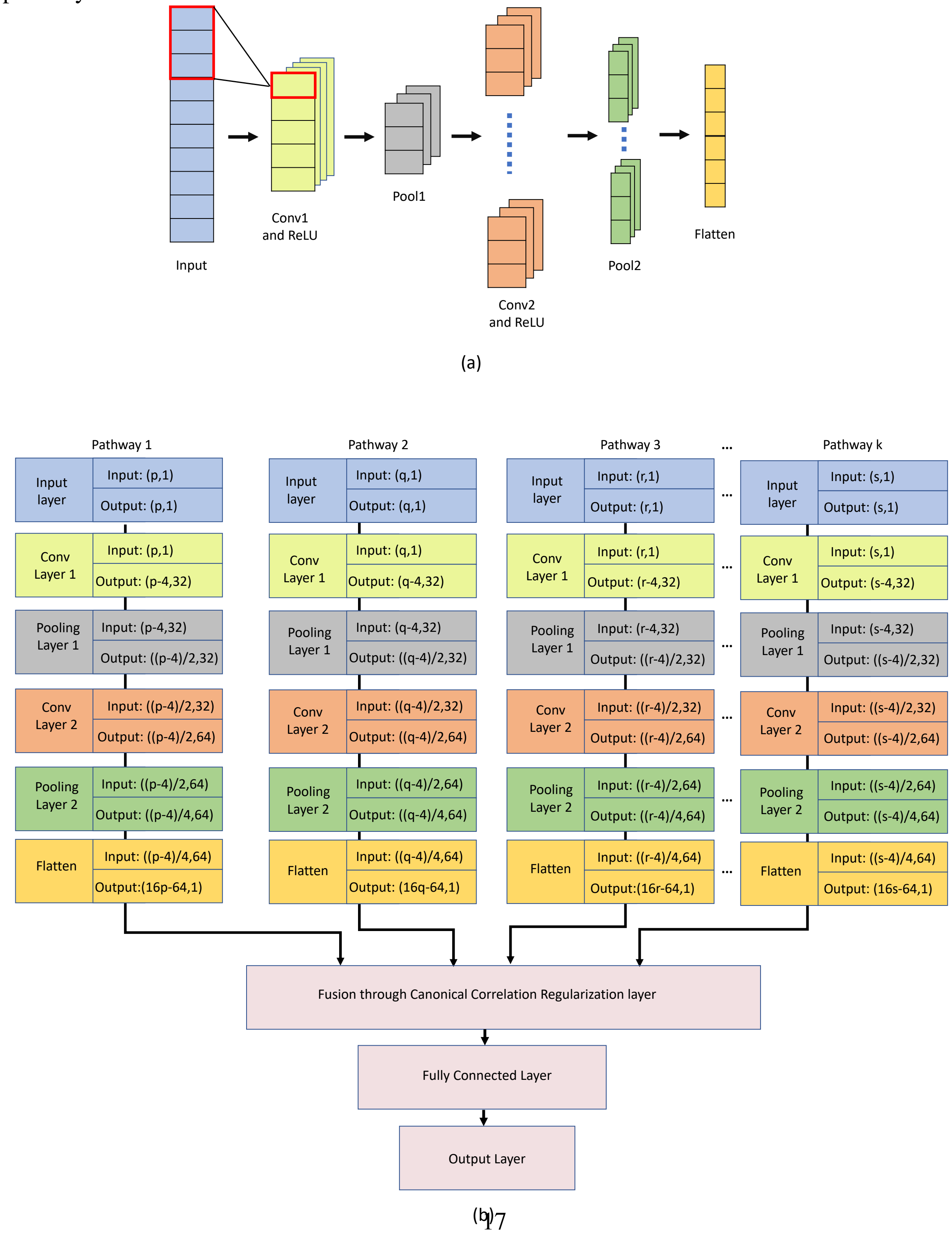

3 References

[1] Liu, J. *et al.* An integrated tcga pan-cancer clinical data resource to drive high-quality survival outcome analytics. *Cell* **173**, 400–416 (2018).
